# Cross-Modal Plasticity during Self-Motion Perception

**DOI:** 10.3390/brainsci13111504

**Published:** 2023-10-24

**Authors:** Rushi Lin, Fu Zeng, Qingjun Wang, Aihua Chen

**Affiliations:** 1 Key Laboratory of Brain Functional Genomics (Ministry of Education), East China Normal University, 3663 Zhongshan Road N., Shanghai 200062, China; 18717751917@163.com (R.L.); 52181300023@stu.ecnu.edu.cn (F.Z.); 52191300023@stu.ecnu.edu.cn (Q.W.); 2 NYU-ECNU Institute of Brain and Cognitive Science, New York University Shanghai, Shanghai 200122, China

**Keywords:** cross-modal, plasticity, self-motion, vestibular, visual

## Abstract

To maintain stable and coherent perception in an ever-changing environment, the brain needs to continuously and dynamically calibrate information from multiple sensory sources, using sensory and non-sensory information in a flexible manner. Here, we review how the vestibular and visual signals are recalibrated during self-motion perception. We illustrate two different types of recalibration: one long-term cross-modal (visual–vestibular) recalibration concerning how multisensory cues recalibrate over time in response to a constant cue discrepancy, and one rapid-term cross-modal (visual–vestibular) recalibration concerning how recent prior stimuli and choices differentially affect subsequent self-motion decisions. In addition, we highlight the neural substrates of long-term visual–vestibular recalibration, with profound differences observed in neuronal recalibration across multisensory cortical areas. We suggest that multisensory recalibration is a complex process in the brain, is modulated by many factors, and requires the coordination of many distinct cortical areas. We hope this review will shed some light on research into the neural circuits of visual–vestibular recalibration and help develop a more generalized theory for cross-modal plasticity.

## 1. Introduction

**Figure 1:**
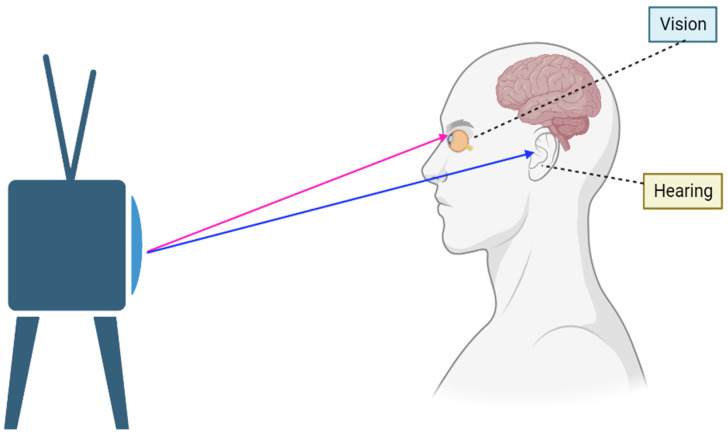
A person watches the TV show by integrating information from sound and sight.

Our world is full of information coded in different sensory modalities, e.g., touch, smell, taste, vision, hearing, vestibular, proprioceptive, and intersensory. Generally, more than one sensory system is activated by an object at the same time, and these signals are transformed, associated, and merged to help us perceive a coherent perception of the world [1,2]. For example, when you watch TV, you see pictures on the screen and hear sounds coming from the speaker to better understand the TV show (Figure 1). Since the information provided by each sense alone is usually noisy or unreliable, combining information across modalities allows one to improve the estimation of objects. Such a process is known as multisensory integration, which requires the sensory cues arising from a common cause to be weighted properly [3]. Multisensory integration is necessary for perception, motor transformation, decision-making, learning, and memory [4]. Deficits in multisensory integration often cause several problems such as dizziness [5]. In addition, some individuals diagnosed with schizophrenia and autism spectrum disorder exhibit multisensory processing failures [6]. Although schizophrenia and autism are distinct diagnoses and their mechanisms are still being explored, the patients share some characteristics in improper multisensory integration: they both integrate multisensory inputs over a longer time-binding window, which leads them to perceive asynchronous auditory and visual events as occurring simultaneously [7,8]. In the past decades, lots of work has focused on how different sensory modalities are integrated to enhance perception and facilitate behavior [9,10,11].

In addition to multisensory integration, another important but usually neglected issue is cross-sensory calibration, where one sense calibrates the other sense rather than fusing with it [12]. For example, vision usually needs to be associated with touch to perceive the physical attributes of “bigness”, since vision is often distorted due to visual distance or angle and has no direct access to “bigness”. Haptic feedback often improves visual perception [13], e.g., young subjects often tend to underestimate the visual size of distant objects, but the visual biases disappear if the subjects are allowed to touch the object [14]. In other words, the touch signals educate (or calibrate) the visual signals; in particular, the ability of children to optimally integrate vision and touch gradually develops up to 8–10 years of age [14,15]. It is worth noting that cross-calibration is not limited to development but is a lifelong process; however, the relevant neural basis has been poorly explored [16].

In this review, we summarize recent work about cross-modal recalibration (mainly based on self-motion perception) and hope to gain some insights about the underlying mechanism and offer some suggestions for future research.

## 2. Cross-Modal Calibration and Recalibration

Due to the external noise caused by dynamic environmental changes and the internal noise of the sensory system, multisensory cues are hardly always kept matched in space and time, and perceptual recalibration is constantly needed to correct discrepancies arising between modalities. As a process of short-term multisensory plasticity, such multisensory calibration is widely observed [17,18,19]. A typical example of cross-modal recalibration is the ventriloquism aftereffect (VAE): during the performance of ventriloquism, the performer manipulates the puppet’s mouth while he/she is speaking, and the audience feels the sound is coming from the puppet rather than the performer. If the auditory and visual stimuli are repeatedly paired with a displaced sound, people will adapt to this ventriloquism illusion. Then, when the visual stimuli disappear, and only the auditory stimulus is presented, the perceived position of the subjects is still shifted to the position of the visual stimulus, indicating that the position of the auditory stimulus is recalibrated during the adaptation to spatially discrepant audiovisual stimuli [20,21,22,23,24,25,26]. Another popular example is RHI (the rubber hand illusion), which results in the proprioception of the hand being offset in the direction of the visually observed rubber hand [27,28,29,30]. Through cross-modal recalibration, the brain corrects conflicts between different modalities and ensures that we maintain a stable and coherent perception of the outside world [31]. However, most research about recalibration is based on behavioral effects [32,33,34]; where and how cross-modal recalibration happens in the brain is still poorly understood.

Until now, only a few studies have focused on the neural mechanism of cross-modal recalibration. Zierul & Bjorn et al. (2017) evaluated the correlation between the cross-modal recalibration of auditory spatial perception and the auditory cortex, using functional magnetic resonance imaging (fMRI). After audiovisual recalibration, the subjects’ auditory spatial perception shifted to the visual position; meanwhile, the BOLD response in the left planum temporale (lPT) was enhanced. The authors considered that cross-modal spatial recalibration could be accomplished by adjusting for single-sensory representations in the primary auditory cortex [35]. However, they only used auditory cues to test the effect, which might underestimate the contributions from other areas (e.g., multisensory area).Later, Park et al. (2021) designed an audiovisual ventriloquism aftereffect experiment, consisting of three sessions: (1) a pre-recalibration session (subjects were given only auditory stimulus), (2) a recalibration session (conflicting combined visual and auditory stimuli were given), (3) post-recalibration session (only auditory stimulus was given). After recalibration, subjects shifted their auditory perception to the visual position, to reduce the audiovisual conflict of position. At the same time, the study recorded the EEG signals from the subjects. During the recalibration session, the parietal region’s EEG activity became significantly different from that of the pre-recalibration session, with increased activity under audiovisual conflict, indicating that the parietal region plays a central role in multi-sensory recalibration [36]. These results suggest that both the primary sensory cortex and parietal cortex are involved in cross-modal recalibration. Recently, Sebastian Scheliga et al. (2022) used activation likelihood estimation (ALE) meta-analysis on the current fMRI literature on multisensory integration to identify a general multisensory interaction network across senses. They found that the bilateral superior temporal gyrus (STG), the middle temporal gyrus (MTG), the thalamus, the right insula, and the left inferior frontal gyrus (IFG) are parts of a common brain network incorporating different functional aspects of multisensory integration, with the thalamus as the first subcortical relay station projecting multisensory information to a higher cortical integration center (the superior temporal gyrus/sulcus, STG/STS), while conflict-processing brain regions such as the insula and inferior frontal gyrus facilitate the integration of incongruent information [37]. However, how these different areas contribute to the multisensory recalibration still needs further investigation. More complicatedly, some brain activity oscillations might be involved in multisensory processing, e.g., Luis mors Fernandez et al. (2018) used EEG to record signals in the anterior cingulate cortex of humans and found that θ oscillations increased under inconsistent audiovisual stimuli [38].

At the single-neuron level, Eric Knudsen et al. (2002) conducted a series of studies on the neural circuitry mechanism of multisensory plasticity in juvenile barn owls: They found the alignment of visual and auditory maps in the optic tectum could be changed adaptively after the young owls wore prismatic spectacles to modify their visual input. The auditory space map was usually recalibrated to align with the shifted visual field. However, when the researchers performed similar operations on owls of different ages, they found that the recalibration was less pronounced in aged than in young owls [39]. Since cross-modal recalibration also widely exists in adults (as mentioned above [40]), a more general neuronal basis for how multisensory systems consistently adapt to each other is necessary.

## 3. Self-Motion Perception Offers a Prime Substrate to Study Cross-Modal Recalibration

Self-motion perception refers to the subjective experience of moving in space, which is crucial for spatial positioning, navigation, and motion planning. It is essentially a multisensory process that relies on vision, vestibular, somatosensory, and other cues [41,42]. Some immersive virtual reality studies have shown that the integration of vision and proprioception is helpful for self-motion encoding [43], while other studies have shown that consistent visual and vestibular signals can also improve self-motion perception [44]. Specifically, optic flow patterns generated by self-motion relative to the stationary environment result in congruent visual–vestibular self-motion signals; however, object motion, vestibular dysfunction, and artificial stimulation can cause incongruent signals. For example, an object moving independently in the world usually alters the optic flow field and may bias heading perception if the visual system cannot dissociate object motion from self-motion. At this time, if adding vestibular self-motion signals to optic flow, vestibular signals can facilitate the dissociation of object motion from self-motion, leading to more accurate heading judgments. As a result, humans and animals usually integrate visual and vestibular signals to guide ongoing movement [44].

As a major contributor to self-motion perception, the vestibular system is highly plastic; for example, patients with central or peripheral unilateral vestibulopathy usually learn to compensate for the vestibular loss (using other senses) with significant functional restoration [45]. Also, if damage to the function of a unilateral vestibular nucleus group results in an imbalance of bilateral vestibular central activity, the contralesional vestibular nucleus will be actively regulated to help restore bilateral vestibular central activity balance, to achieve vestibular compensation [46]. On the other hand, the vestibular sensation often shows dynamic recalibration in altered states such as the ocean or space [47,48]. For example, when astronauts enter space, they usually develop space motion sickness symptoms, which abate after several days, and they adapt to the microgravity environment within a few days. After returning to the ground at the end of a mission, astronauts again experience sickness and need some time to readapt to the Earth’s 1G environment. Furthermore, vestibular motion often leads to adaptive aftereffects [49,50,51], e.g., adaptation to a short-term (2–10 s) rotation at a relatively slow (10–60 °/s) speed in the horizontal plane usually leads to changes in perception of the subsequent test rotations [51].

In addition, self-motion perception shows a high degree of flexibility to the perturbations of sensory statistics. For example, Fetsch et al. (2009) trained monkeys to perform a heading discrimination task using optic flow (visual condition), inertial motion (vestibular condition), or a combination of both cues (combined condition). For two-thirds of combined trials, a small conflict angle (e.g., +4°, −4°, or 0°) was interposed between the visual and vestibular heading trajectories. Cue reliability was varied randomly across trials by changing the motion coherence of the optic flow stimulus. The study found that when visual cue reliability was low, the psychometric function during cue conflict shifted in the direction that indicates vestibular dominance. When visual reliability was high, the curves shifted in the opposite direction, indicating visual dominance. Thus, monkeys can dynamically adjust their cue weights on a trial-by-trial basis, indicating that the process of multisensory integration is plasticity [52].

In particular, several types of visual–vestibular recalibration have been characterized during self-motion perception: long-term recalibration involving perceptual adaption to a mismatching visual–vestibular signal [53,54] and rapid recalibration about how recent prior stimuli and choices affect subsequent self-motion decisions [55].

### 3.1. Long-Term Visual–Vestibular Recalibration

Long-term visual–vestibular recalibration refers to the recalibration due to the presence of a consistent discrepancy (usually requires approximately 1 h) between visual and vestibular signals, e.g., the experiments carried out by Zaidel et al. (2011). The experimental session consisted of three consecutive blocks: (1) A pre-recalibration block comprising cues from only a single (visual only/vestibular only) modality, interleaved. They used this block to deduce the baseline bias and individual reliability of each modality for the subjects; during each trial, the subject experienced real or simulated translational motion in the horizontal plane and reported the perceived direction of motion (rightward vs. leftward relative to straight ahead). (2) A recalibration block: In this block, only combined visual–vestibular cues were presented. For all the trials, a fixed discrepancy between the visual and vestibular cues was introduced. (3) A post-recalibration block: During this block, the calibration of the individual (visual/vestibular) modalities was measured by single modality trials (as in the pre-calibration block) interleaved with the cue combination trials. The cue combination trials were required to maintain the calibration during measurement. By comparing the post-adaption block to the pre-adaption block, the study found that visual and vestibular cues significantly adapted in the direction required to reduce cue conflict [53]. In the absence of external feedback, the vestibular adaptation was greater than the visual adaptation, with a ratio of vestibular/visual adaptation of about 2:1 for both humans and monkeys [53]. For cross-modal recalibration, there are several theoretical models: one is the visual-dominant recalibration model, which states that only non-visual information will recalibrate visual information [56]; the other is the reliability-based recalibration model, which states that multisensory recalibration is determined by the relative reliability (also known as precision, meaning that repeated exposure to the same stimulus repeatedly yields the same percept consistently) of each cue [32,34]. Zaidel et al. (2011) quantitatively investigated whether the visual–vestibular recalibration was reliability-based or visual-dominant, and they found that it could be described best with a model of fixed-ratio adaptation (visual-dominant adaptation is only a subcase of a generalized fixed-ratio adaptation model) [53], regardless of relative cue reliability.

Since the most reliable cue might not always be the most accurate, the study further examined whether the ratio of adaptation changed with cue accuracy. In a follow-up study, the researchers tested a supervised self-motion recalibration by providing feedback on accuracy [54]. They still used a visual–vestibular version of the ventriloquism aftereffect, which consisted of three consecutive blocks: pre-recalibration, recalibration, and post-recalibration. However, in the recalibration block, the monkeys were presented with combined stimuli (simultaneous visual and vestibular cues) with a systematic discrepancy between the visual and vestibular heading directions; the reward was consistently contingent on one of the cues (visual or vestibular). The reward-contingent cue was considered externally accurate. The study found that supervised recalibration is a high-level cognitive process that compares the combined-cue (multisensory) estimate to feedback from the environment. This results in a “yoked” recalibration of both cues in the same direction, to reduce conflict between the combined estimate and external feedback [54]. Thus, both the feedback and reliability affect the supervised visual–vestibular recalibration, and the study claimed that the unsupervised and supervised recalibration might work in parallel to ultimately achieve the optimal solution of both internal consistency and external accuracy.

### 3.2. Short-Term Visual–Vestibular Recalibration

Although most studies on multisensory recalibration have focused on the adaptation to consistent discrepancies in the sensory inputs, we seldom meet such long-term (from minutes to hours) systematic sensory discrepancies during our daily lives. Thus, some researchers have tried to investigate short-term recalibration. Cuturi et al. (2014) examined whether subjects’ vestibular perception in darkness was affected after exposure to a sustained visual stimulus. Specifically, researchers asked subjects to judge the direction of vestibular movement in a dark state after giving them a prolonged visual stimulus; they found relatively long (≥15 s) visual self-motion stimuli can bias the subsequent vestibular movement judgment, and that shorter duration (e.g., 1.5 s, 3.75 s, and 7.5 s) stimuli do not elicit cross-sensory (visual↔vestibular) adaptation [57,58]. However, Shalom-Sperber et al. (2022) found that cross-sensory (visual→vestibular or vestibular→visual) adaptation occurred after experiencing several short (1 s) self-motion stimuli. In their experiment, they grouped trials in batches, and each batch comprised three “prior” trials (headings biased to the right or left) followed by one “test” trial (unbiased “test” trial). Right- and left-biased batches were interleaved pseudorandomly. Significant adaptation was observed in both cross-sensory conditions (visual prior and vestibular test trials, and vice versa), as well as both unisensory conditions (when prior and test trials were of the same modality, either visual or vestibular). By fitting the data with a logistic regression model, the study found that adaptation was elicited by the prior stimuli (not prior choices). These results suggest that the brain monitors supra-modal statistics of events in the environment, even for short-duration stimuli, leading to functional (supramodal) adaptation of perception. A possible reason for the difference between Cuturi’s study and Shalom-Sperber’s study might be whether the prior stimulus (or “adaptive stimulus”) is judged, since in Cuturi et al.’s study the subjects did not judge the “adaptive” stimulus while in Shalom-Sperber’s experiment subjects needed to judge the priori stimuli [55]. Thus, short-term visual–vestibular recalibration usually is not only affected by cue reliability and accuracy feedback (or prior belief) but is also affected by recent history (prior stimuli and choices). As a result, short-term visual–vestibular recalibration is a high-level multisensory plasticity.

## 4. Neural Correlates of Visual–Vestibular Recalibration

During the past decades, substantial progress has been made in the neural mechanism of visual–vestibular interaction in the cortex. Several cortical areas have been reported to have robust responses to visual and vestibular self-motion stimuli, including the dorsal medial superior temporal area (MSTd) [10], the ventral intraparietal area (VIP) [59], the visual posterior sylvian area (VPS) [60], the frontal eye field (FEF) [61], and the superior temporal polysensory area (STPp) [62]. Among these areas, both MSTd and VIP were reported to show high correlations with multisensory integration heading discrimination tasks [11,63]. However, MSTd inactivation had strong effects on visual heading discrimination and significant but weak effects on vestibular heading discrimination [64], while large bilateral muscimol injections into VIP revealed no deficits in performance [65]. Further analysis revealed that tuning in MSTd neurons primarily reflects sensory inputs [66], while VIP is marked by strong choice signals and is considered a higher-level multisensory area, possibly involved in perceptual decision-making or higher-order perceptual functions [65,66,67].

Since MSTd is an important area for visual–vestibular integration, it is also considered to play an important role during visual–vestibular multisensory plasticity. In a previous study, Morgan et al. (2008) evaluated the combination rules employed by multisensory neurons in MSTd: they found that when visual stimuli were degraded, visual weights for multisensory neurons in MSTd decreased, and vestibular weights increased [68]. In other words, weights can change with the relative reliabilities of the two cues during multisensory stimulation, which is a sign of multisensory plasticity. Later, Fetsch et al. (2009) found that monkeys and humans can dynamically adjust their cue weights on a trial-by-trial basis in a heading discrimination task involving visual and vestibular cues [52]; they then searched the neural correlates of reliability-based cue weighting during multisensory integration [69]. They recorded extracellular single-unit activity in MSTd during the heading discrimination task and found that the activity of multisensory neurons in MSTd is also modulated by changes in cue reliability across trials, indicating that MSTd might contribute to visual–vestibular plasticity.

**Figure 2:**
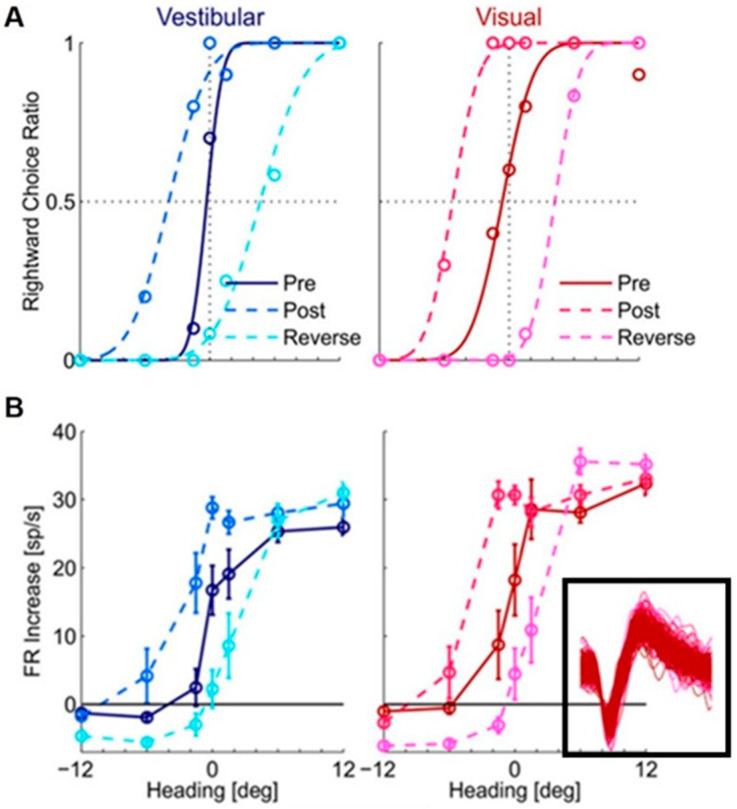
Shifts in (**A**) monkey’s behavior and (**B**) tuning of a VIP neuron during the supervised visual–vestibular conflict calibration. Blue and red represent vestibular and visual responses, respectively. Pre-calibration is indicated by dark blue (vestibular) and dark red (visual), and post-calibration is indicated by light blue (vestibular) and light red (visual). Cyan and magenta represent the vestibular and visual responses after calibration in the reverse direction. For behavior (**A**), circles show the proportion of rightward choices (fit by cumulative Gaussian psychometric curves). For the neuronal responses (**B**), circles and error bars show mean FR (baseline subtracted) ± SEM. The inset shows one hundred (randomly selected) overlaid spikes from each block, indicating these spikes are from the same neuron. “Reprinted with permission from [70]. 2021 Adam Zaidel”.

However, when Zaidel et al. (2021) used a visual–vestibular version of the ventriloquism aftereffect to search the neuronal substrate of supervised recalibration, they found a strong neuronal recalibration in VIP but not MSTd. The protocol consisted of the three consecutive blocks mentioned before: pre-recalibration, recalibration, and post-recalibration. The neuronal activities were recorded in VIP and MSTd when the monkeys performed the task, then the difference in the neuronal tuning to vestibular or visual signals between the pre-recalibration period and post-recalibration period was compared. The study found that the neuronal tuning of both vestibular and visual signals in VIP shifted together with the behavior recalibration [70] (Figure 2). In other words, both vestibular and visual cues were “yoked” in the same direction during the supervised recalibration; the neuronal tuning also shifted in the same direction, which resulted in undetectable differential aspects of neuronal recalibration for the individual cues.

**Figure 3:**
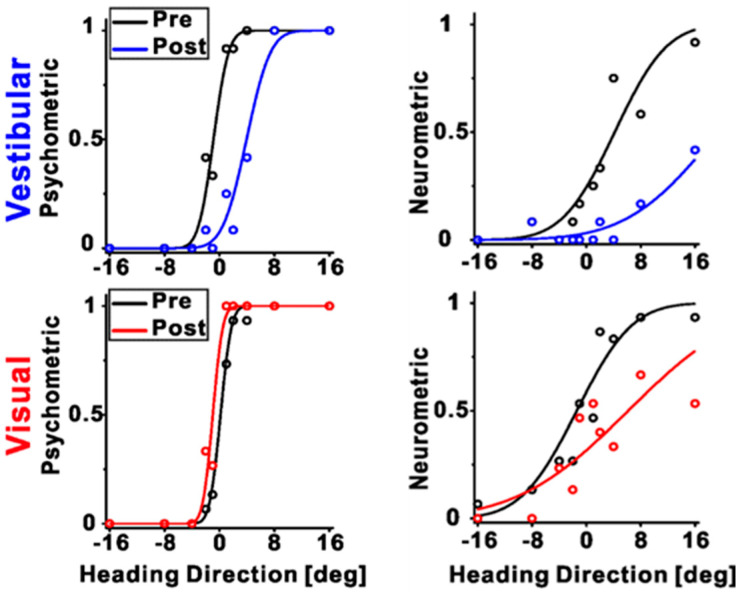
An example session during unsupervised recalibration with simultaneous recording from VIP. The left panels depict the behavioral responses, pre- (black color), and post-recalibration (red color for vestibular condition and blue color for visual condition, respectively). The right panels show the corresponding neurometric curves with fitted cumulative Gaussian functions. Each data point shows the proportion of trials in which an ideal observer would make a rightward choice given the FRs of the neurons. For this example session, the vestibular neurometric curve shifted rightward, in accordance with the vestibular perceptual shift. Interestingly, the visual neurometric curve also shifted rightward, although the visual perceptual shifted leftward. “Reprinted with permission from [71]. 2023 Fu Zeng”.

**Figure 4:**
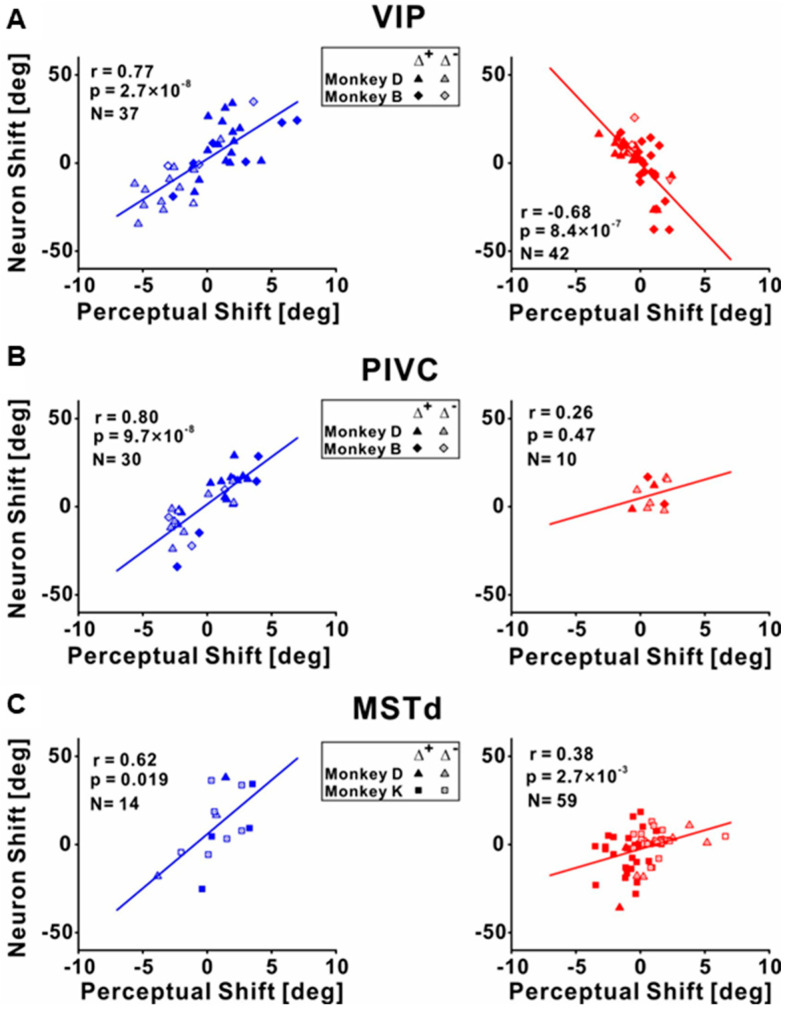
Correlations between the neuronal tuning shift and behavior shifts during unsupervised recalibration. (**A**) Correlations between neuronal shifts and perceptual shifts for the vestibular (left panel) and visual cues (right panel) in VIP. The shifts were quantified by the difference between the post- vs. pre-recalibration curves’ PSEs (points of subjective equality, as shown in Figure 2 or Figure 3). The left panel shows the vestibular condition, and the neuronal shifts were positively correlated with the behavior shifts. The right panel shows the visual condition, and the neuronal and perceptual shifts were negatively correlated. (**B**) Significant positive correlations between neuronal PSE shifts and perceptual PSE shifts for the vestibular and visual cues in PIVC. (**C**) Significant positive correlations between neuronal PSE shifts and perceptual PSE shifts for the vestibular and visual cues in MSTd. “Reprinted with permission from [71]. 2023 Fu Zeng”.

To investigate the differences between the individual cues recalibrated in the brain, Zeng et al. (2023) recorded the neuronal activities among different multisensory cortical regions during unsupervised recalibration (monkeys were not required to choose in the recalibration period [53]). This unsupervised paradigm elicits behavioral shifts in the opposite direction, and thus can more readily discern if the vestibular neurometrics shift with visual (rather than vestibular) behavioral shifts. They found that vestibular tuning in VIP recalibrated with vestibular perception; surprisingly, visual tuning shifted contrary to visual perceptual shifts (and rather in accordance with vestibular recalibration) (Figure 3 and Figure 4A). Since unsupervised recalibration occurs in the absence of external feedback, it was thought to reflect an implicit change in perception, with effects relatively early in the visual–vestibular integration hierarchy and then propagating to higher-level areas. The study also examined two relatively early multisensory cortical areas, MSTd (dominated by visual signals) [10] and PIVC (the parietal insular vestibular cortex, dominated by vestibular signals) [65]. The results were very different from VIP. Specifically, in PIVC, vestibular tuning similarly shifted in the same direction as vestibular perceptual shifts (the PIVC cells were not robustly tuned to visual stimuli) (Figure 4B). In MSTd, both neuronal MSTd responses to vestibular and visual cues shifted according to their respective cues’ perceptual shifts. (Figure 4C). Thus, the VIP visual response is not a simple feedforward projection from the early visual area MSTd dominated by sensory signals. However, the origin of the visual recalibration signals in VIP remains unknown. Overall, these results reveal profound differences in neuronal recalibration across multisensory cortical areas, indicating different functions across these areas.

The above results also suggest that multisensory neuronal recalibration is more complicated than previously thought [59,71], and the neural circuity underlying the visual–vestibular recalibration needs further investigation. As we addressed before, at the behavior level, visual–vestibular recalibration is not a passive experience simply driven by bottom-up sensory signals, but can be affected by lots of factors: the supervised recalibration experiments demonstrated that the reward signals as accuracy feedback can affect the recalibration, while the short-term recalibration revealed that the individual’s prior experience or choice also affects the individual’s multisensory recalibration. At the neurophysiology level, how the visual–vestibular recalibration is encoded and decoded remains unknown. Until now, only a few areas have been investigated; however, multisensory interaction occurs all along the brain hierarchy with specific functions at different stages [72]. In particular, some subcortical areas are also involved in multisensory signal processing, e.g., superior colliculus (for review, see Stein et al., 2008; [73]) and zona incerta (ZI, Shen et al., 2019 [74]). In addition, the self-motion pathway is shared with touch signals [75], and multisensory processing is part of the sensorimotor integration loops with pathways. As a result, these sensory signals have to be explained by considering sensorimotor context. Accordingly, the cerebellum might also be of interest for visual–vestibular recalibration, since it can adapt to different sensory streams depending on the behavioral context [76]. Thus, more areas need to be investigated to see whether they are involved during visual–vestibular recalibration, and the specific roles of each area also need to be examined. In addition, the whole network and the underlying neural circuits of how these different areas interact with each other during visual–vestibular recalibration also need to be considered.

## 5. Summary and Future Directions

Cross-modal recalibration is important for us to survive in a dynamically changing environment. Here, we reviewed different types of visual–vestibular recalibration during self-motion perception. Specifically, we discussed the neural mechanism of long-term recalibration due to discrepant visual and vestibular stimuli, and exposed profound differences in neuronal recalibration among different multisensory cortical areas. However, the exact neural mechanism of long-term recalibration is still not clear, which might require other areas to be investigated. In particular, the causal connections between these areas and the recalibration behaviors need further exploration.

On the other hand, since we interact with our environment using sequences of actions dealing with different stimuli, short-term recalibration occurs more often. Whether the short-term recalibration shares the same neural underpinnings as long-term recalibration still needs further investigation.

## Data Availability

Not applicable.
